# Overweight in Adolescence Can Be Predicted at Age 6 Years: A CART Analysis in German Cohorts

**DOI:** 10.1371/journal.pone.0093581

**Published:** 2014-03-27

**Authors:** Christina Riedel, Rüdiger von Kries, Anette E. Buyken, Katharina Diethelm, Thomas Keil, Linus Grabenhenrich, Manfred J. Müller, Sandra Plachta-Danielzik

**Affiliations:** 1 Ludwig-Maximilians University of Munich, Institute of Social Paediatrics and Adolescent Medicine, Munich, Germany; 2 IEL-Nutritional Epidemiology, University of Bonn, DONALD Study at the Research Institute of Child Nutrition, Dortmund, Germany; 3 Institute of Social Medicine, Epidemiology and Health Economics, Charité Universitätsmedizin Berlin, Berlin, Germany; 4 Christian-Albrechts University of Kiel, Institute of Human Nutrition and Food Science, Kiel, Germany; Scientific Directorate, Bambino Hospital, Italy

## Abstract

**Objective:**

To examine, whether overweight in adolescents can be predicted from the body mass index (BMI) category, at the age of 6, the mother's education level and mother's obesity and to quantify the proportion of overweight at the age of 14 that can be explained by these predictors.

**Method:**

Pooled data from three German cohorts providing anthropometric and other relevant data to a total of 1 287 children. We used a classification and regression tree (CART) approach to identify the contribution of BMI category at the age of 6 (obese: BMI>97^th^ percentile (P97); overweight: P90<BMI≤P97; high normal weight: P75<BMI≤P90; third quartile: P50<BMI≤P75; below the median: ≤P50), maternal education level and maternal obesity for prediction of overweight/obesity (BMI>P90) at the age of 14.

**Results:**

While 4.8% [95%CI: 3.2;7.0] of 651 boys and 4.1% [95%CI: 2.6;6.2] of 636 girls with a BMI<P75 at age 6 were overweight/obese in adolescence, prevalence increased to 41.3% [95%CI: 31.9;51.1] and 42.5% [95%CI: 33.8;51.6], respectively, in those with BMI≥P75. The lowest prevalence was 1.9% [95%CI: 0.8;3.8] in boys with a BMI≤P50 and the highest prevalence 91.7% [95%CI: 61.5;99.8] with a BMI>P97 (similar results for girls). BMI≥P75 at the age of 6 explained 63.5% [95%CI: 51.1;74.5]) and 72.0% [95%CI: 60.4;81.8] of overweight/obesity at the age of 14 in boys and girls, respectively.

**Conclusions:**

Overweight/obesity in adolescence can be predicted by BMI category at the age of 6 allowing for parent counselling or risk guided interventions in children with BMI≥P75, who accounted for >2/3 of overweight/obesity in adolescents.

## Introduction

Childhood obesity is an increasing and challenging problem [1], [2], [3], [4], [5] due to short-term consequences especially regarding psychosocial effects for the adolescents [6] and long-term health risks like non-insulin-dependent diabetes mellitus, sustained hypertension, and other cardiovascular conditions [2], [6], [7]. The development of effective prevention strategies requires the understanding of the natural life course of obesity in children [8]. Primary school age has recently been identified as a critical period for the emergence of overweight [9], [10], [11], [12], [13], [14]. Overweight/obesity in early primary school years was identified as an important predictor for later overweight because of low remission rates [9], [10], [15], [16], [17]. Overweight/obesity in early primary school years however, accounted only for a small proportion of overweight in adolescence and young adults because of a low population attributable risk: a substantial proportion of overweight/obese adolescents was not overweight/obese in early primary school years [15], [16], [18].

There are some studies pointing to varying contributions for incident overweight/obesity by different normal weight body mass index (BMI) categories [19], [20], [21]. In order to identify high risk groups for early targeted prevention or therapy of later overweight/obesity we used a classification and regression tree (CART) approach to develop a simple binary tree-structured decision algorithm [22], [23] for later overweight/obesity by different BMI categories at the age of six years, mothers' obesity and education status. Can the BMI category at the age of six years with or without additional information on the mother's education level and obesity be used to help optimizing targeted preventive or therapeutic interventions for overweight/obesity in young children as advocated previously [24]. What is the proportion of overweight /obesity in adolescence predicted by these risk factors?

## Methods

### Ethics Statement

All cohort studies had obtained ethical approval by their local ethics committee.

### Study Population and Data Sources

We merged three German cohorts providing carefully measured anthropometrical data of weight and height of children between the ages of 6 to 14 years on 1 287 children. The Kiel Obesity Prevention Study (KOPS) is a cluster randomised intervention study which was undertaken between 1996 and 2001 in the context of school entry health examination (SEH) [9], [25]. A representative group of 4 997 children participated in the study [25], [26]. Follow-up information was collected during examinations performed in the school setting between 2000 and 2005 at the 4^th^ and between 2004 and 2010 at the 8^th^ grade where 1 671 and 748 children of the original population (15% of the origin and 45% of the population of the 4 year follow-up, respectively) participated [9], [25]. From these 748 children 601 did not take part in a school intervention program. Compared to the original population fewer children were overweight and more were from higher educated families [25]. Measurements of height and weight were performed by trained nutritionists and physicians [27]. Weight and height at birth and at two years was abstracted from the baby checkup booklets. Mothers' self-reported weight and height and education status were ascertained from a self administered questionnaire. Data for 494 children with complete information on BMI at the age of 2, 6 and 14 years and maternal education and maternal obesity were available.

The Dortmund Nutritional and Anthropometric Longitudinally Designed (DONALD) Study is a longitudinal open cohort study [28], [29]. Since 1985, about 35-40 newborns are annually recruited into the study examined at ages 3, 6, 9, 12 and 18 months and annually thereafter until young adulthood [28]. The elaborate design of the DONALD Study results in an over-representation of children from families with high socioeconomic status. However, BMI values of DONALD participants between the ages 2 and 18 are similarly distributed when compared to the standard German reference data [10], [30]. Children were measured by trained nurses [29]. On admission, parents are interviewed about familial characteristics and are weighed and measured. 379 children had anthropometric measurement taken at 2, 6 and 14/15 years.

The German Multicenter Allergy Study (MAS) was launched in 1990, prospectively assessing development of allergic diseases and potential exposures, including size and weight, in 5 German cities (Berlin, Düsseldorf, Freiburg, Mainz, Munich) [31], [32], [33]. Of 1 314 recruited newborns (499 with family history of allergy) were followed up at 1, 3, 6, 12, 18, and 24 months and then annually until the age of 13. 721 (54.9%) of the enrolled children were examined at the age of 13. The MAS cohort had standardized physical examinations by physicians [31], [34]. 414 children with anthropometric measurement taken at 2, 6 and 13 years and information on maternal BMI and education status were included.

In total 1 287 children were merged from the three cohorts.

### Definition of Main Outcome Variable

Overweight/obesity (BMI>90^th^ percentile (P90)) at the age of 14 years, classified according to age- and sex-specific German reference percentiles [30].

### Explanatory Variables

The explanatory variables were: BMI category at the age of 6 years: obese: BMI>P97; overweight: P90<BMI≤P97; high normal weight: P75<BMI≤P90; third quartile: P50<BMI≤P75; below the median: ≤P50 according to age- and sex-specific German reference percentiles [30]; maternal education defined as high for 10 years of school education or above and as low with less than 10 years of school education; maternal weight status categorized as obese (BMI≥30) or not obese (and BMI<30). We considered maternal obesity and education which is a stronger determinant for offspring BMI or overweight than paternal obesity and education, and maternal obesity instead of maternal overweight because maternal obesity is a stronger risk factor for childhood overweight than maternal overweight [20], [35].

### Statistical Analysis

We used analysis of variance (ANOVA) models for comparisons of numeric characteristics of all studies. Differences between proportions were tested by a test of equal proportions [36].

We used the classification tree approach to identify the contribution of BMI category at the age of 6 (ordinal scale) and of maternal education level (binary scale) and maternal obesity (binary scale) for overweight/obesity at the age of 14 years [37]. Since previous studies found gender specific differences [15], [17] we calculated the CARTs separately for boys and girls.

For the analysis which is based on repetitive binary splits, the best binary splits were identified from the ordinal scale. The deviance split criteria proposed by Clark and Pregibon [38] for binary classification was applied [39]. The Receiver Operating Characteristic (ROCs) and the respective area under the curve (AUC) were estimated. A sensitivity analysis was performed based on the respective percentiles generated with the revised BMI LMS coefficients by Cole and Lobstein [40] corresponding to the international (International Obesity Task Force; IOTF) cut-offs. These LMS coefficients are based on BMI data of six countries: USA, Britain, the Netherlands, Singapore, Hong Kong and Brazil. These percentiles were applied to categorize the BMI values at the age of 6 (BMI>P97, P90<BMI≤P97, P75<BMI≤P90, P50<BMI≤P75, BMI≤P50) and 14 years (BMI>P90) as observed and the respective CART was calculated. In order to assess the proportion of overweight at the age of 14 explained by BMI category at the age of 6, the proportions of overweight adolescents in the respective categories was determined and compared. For each proportion the exact 95% clopper-pearson confidence intervals were calculated [41].

All calculations were carried out with the statistical software R 2.14.2 (http://cran.r-project.org), using the *stat* and *tree* packages.

## Results

Mean age at the time points considered for life course analysis and mean BMI at the respective time points were similar in the three cohorts with the exception of MAS contributing data for 13 year old children accounting for lower BMI values (Table 1). Gender, gestational age, birth weight of the children and the proportion of obese mothers in the three cohorts was almost identical. The proportion of mothers with the lowest educational level was higher in MAS and lower in DONALD.

**Table 1:** Participant characteristics of the 3 German children cohorts included in the combined data analyses.

Child/ parental characteristics	Merged dataset	KOPS	MAS	DONALD	P value^c^
	N = 1 287	n = 494	n = 414	n = 379	
Gender: male, %	50.6%	47.6%^b^	53.6%^b^	51.2%^b^	0.18
Year of birth, range	1985–1998	1990–1996	1990	1985–1998	
Gestational age, mean (SD)	39.7 (1.6)	39.5 (1.6)^a^	40.0 (1.5)^a^	39.7 (1.5)^a^	P<.001
Birth weight, mean (SD), g	3 421 (515)	3 426 (570)^a^	3 401 (446)^a^	3 435 (511)^a^	0.62
Maternal obesity, %	6.1%	7.3%^b^	4.1%^b^	6.9%^b^	0.11
Mother's educational achievement less	17.5%	17.2%^b^	25.4%^b^	9.2%^b^	P<.001
than 10 years of school education, %					
BMI at 6 years, mean (SD), kg/m^2^	15.5 (1.6)	15.5 (1.6)^a^	15.6 (1.7)^a^	15.5 (1.4)^a^	0.54
BMI at 13/14 years, mean (SD), kg/m^2^	20.3 (3.3)	20.6 (3.1)^a^	19.7 (3.6)^a^	20.1 (3.2)^a^	P<.001
Age at 6, mean (SD), years	6.1 (0.3)	6.3 (0.4)^a^	6.0 (0.1)^a^	6.0 (0.1)^a^	P<.001
Age at 13/14, mean (SD), years	14.1 (0.8)	14.6 (0.5)^a^	13.1 (0.3)^a^	14.5 (0.5)^a^	P<.001

a ANOVA for test of equality of means.

b Test for equality of proportions.

c P values for comparison of characteristics of KOPS, MAS and DONALD.

**Figure 1:**
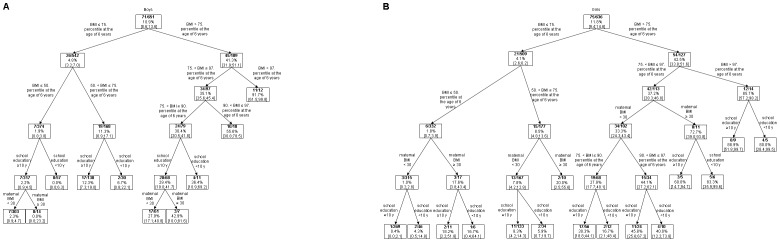
Classification tree for overweight/obese boys (Panel A) and girls (Panel B) at the age of 14 years. Classification tree for overweight/obese boys (Panel A) and girls (Panel B) at the age of 14 years. by different BMI categories at the age of 6, maternal obesity and education level. The numbers represent the number of overweight/obese boys and the total number in each knot as well as the prevalence of overweight/obesity in adolescence with 95% CI's.

Figure 1A depicts the classification tree for overweight at the age of 14 years in boys. The numbers represent the number of overweight/obese girls and the total number in each knot as well as the prevalence of overweight/obesity in adolescence with 95% CI's. The first split divided the five BMI categories at the age of 6 at BMI>P75 or below. Among boys with a BMI>P75 at the age of 6 years 41.3% [95%CI: 31.9; 51.1] were overweight/obese in adolescence whereas only 4.8% [95%CI: 3.2;7.0] of the boys with BMI≤P75 became overweight. Subsequent splits by BMI categories yielded the lowest prevalence (1.9% [95%CI: 0.8;3.8]) in boys with a BMI≤P50 and the highest prevalence (91.7 [95%CI: 61.5; 99.8]) with an BMI>P97. In girls and boys almost the same splits were observed at BMI P75 and for BMI≤P75. In contrast, between P97 und P75 a split by maternal BMI<30 preceded the split of BMI category P90<BMI≤P97 and P75<BMI≤P90 in girls but not in boys (Figure 1B). In both genders further splits for maternal education did not yield additional information. The classification trees based on BMI category, maternal obesity and education had an area under the curve of 0.83 [95%CI: 0.76;0.86] in boys and 0.83 [95%CI: 0.78;0.87] in girls, respectively, indicating fair accuracy. Only marginal changes of the AUC could be detected if the classification trees were based on BMI category at the age of 6 years alone. A sensitivity analyses based on percentiles derived from the IOTF reference population showed very similar results (Figure S1 and Figure S2). As with the age- and sex-specific German reference percentiles, the first split was at BMI>P75 using IOTF. Most subsequent splits were identical as well. Applying these percentiles the prevalence of overweight at the age of 14 years was higher in both genders explainable due to lower BMI cut-off values at the respective P90 (e.g. P90 in 14 year old males, Germany: 23.72; IOTF: 22.48).

**Figure 2:**
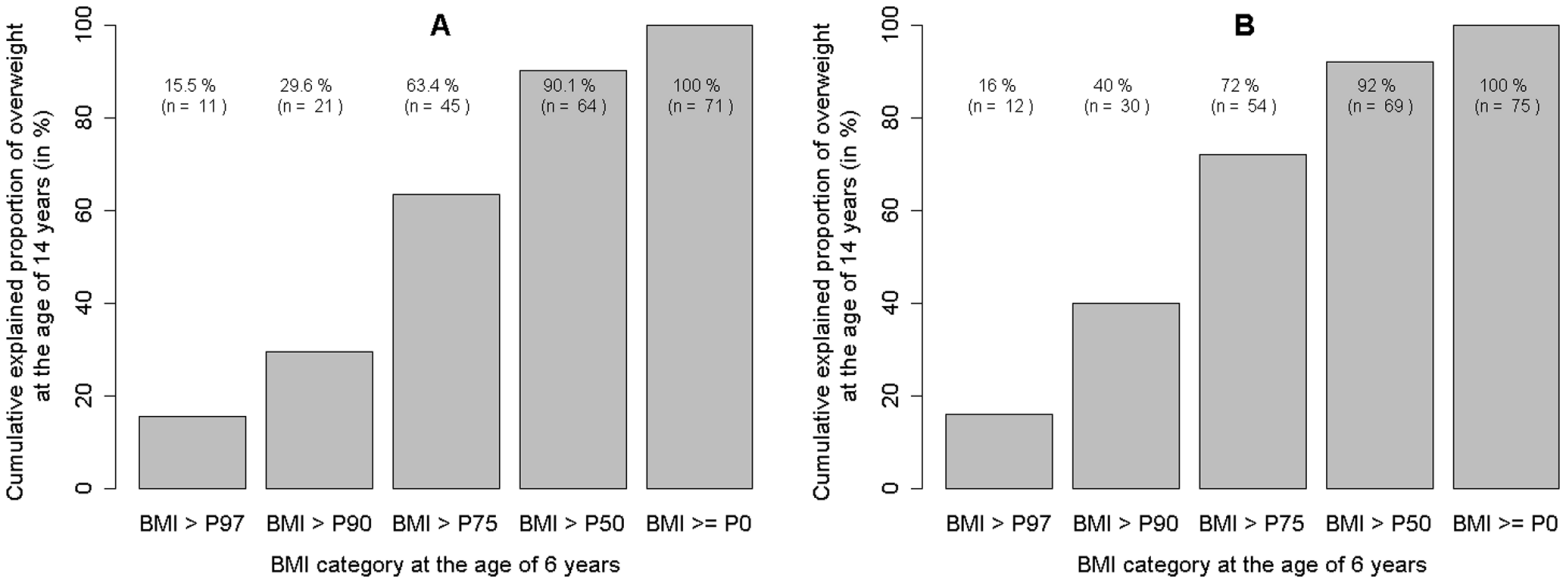
Cumulative explained proportions of overweight/obesity at the age of 14 years. Cumulative explained proportions of overweight/obesity at the age of 14 years by different BMI categories at the age of 6 years in 71 overweight/obese boys (Panel A) and 75 overweight/obese girls (Panel B) in adolescence. The numbers show the cumulative number of boys and girls that are overweight/obesity at the age of 14 years (e.g. 64 boys who are overweight/obese at the age of 14 years lie in the group of BMI >P50 at the age of 6 years; only 7 boys who are overweight/obese at the age of 14 years (71–64) had a BMI≤P50 at the age of 6 years).

Figure 2A and B show the cumulative proportions of overweight/obesity in 71 and 75 overweight/obese adolescent boys and girls, explained by different BMI categories at the age of 6 years. The numbers show the cumulative number of boys that are overweight/obesity at the age of 14 years. In boys overweight or obesity at the age of 6 combined explained only 29.6% [95%CI: 19.3;41.6] of risk of overweight/obesity in adolescence whereas BMI above P75 explained 63.5% [95%CI: 51.1;74.5]) due to the considerable contribution by high normal weight children. The respective figures in girls were 40.0% [95%CI: 28.9;51.7] for overweight/obesity and 72.0% [95%CI: 60.4;81.8] for BMI above P75. In contrast, the children with a BMI below P50 (n = 374 boys and n = 332 girls) at the age of 6 years contributed only 9.9% [95%CI: 4.1;19.3] and 8.0% [95%CI: 3.0;16.7] of overweight/obesity among adolescent boys and girls, respectively. Additional consideration of maternal obesity only marginally improved the explained proportion of overweight/obesity in adolescence (data not shown).

## Discussion

### Principal Findings

The BMI status at the age of 6 years was an important predictor for overweight/obesity in adolescence. In a population with an overall risk of overweight/obesity of 11.3% at age 13/14 years, only few boys and girls with a BMI<P75 became overweight/obese in adolescence. The risk for those with a BMI≥P75 was >40% in both genders, increasing to almost 90% if they were obese at the age of 6 years. About 2/3 of the adolescents with overweight/obesity at the age of 14 years had a BMI above P75 at the age of 6 years. Therefore a risk based strategy for obesity prevention and therapy based on BMI at the age of 6 years might be warranted.

### Comparison with Other Studies

Prediction of later overweight/obesity by BMI category in children has been subject to a number of studies [15], [42], [43], [44] and at least one meta-analysis [13]. In general good prediction was observed which is likely to reflect fair tracking of BMI over the life course [45]. There are, however, only few studies assessing the prediction of later overweight/obesity by overweight in early primary school years [9], [10], [11], [12], [13], [14] and even fewer considering prediction by different BMI categories within the range of normal weight [19], [20], [21]. While confirming that most overweight or obese children in early primary school years remain overweight/obese because of low remission rates [9], [10], [15], [16], [17], our data point to the importance of high normal weight as an additional important predictor for later overweight/obesity, which has already been suggested previously [19], [20], [21]. High normal weight and overweight/obesity combined in early primary school years explained about 2/3 of overweight/obesity in adolescence. Identification of children at high risk for later overweight/obesity at the onset of a life period, when the incidence of overweight/obesity is high [9], [11], may help tagging a high risk group during a critical time window during the obesity life course.

To our knowledge this is the first study using the CART approach to assess the prediction of overweight/obesity in adolescence from BMI category at the age of six years. Compared to the frequently reported relative risks, which by definition refer to reference categories the CART approach describes absolute risks, allowing to identify the risk for the outcome of interest in differently exposed individuals. This CART model splits on independent variables that best predict overweight/obesity in the adolescence and thus identifies the variables most important for the risk of becoming overweight/obese in adolescence [46]. A further advantage is the possibility to handle nominal, ordinal and metric independent variables, [47] and accuracy in the prediction [23]. Although maternal obesity and education are established risk factors for overweight/obesity in the offspring [9], [20], [44] additional inclusion of these risk factors yielded almost identical results. Although this might appear surprising with respect to the high odds ratios reported for maternal obesity and education in analyses (e.g. [48], [49]) which did not consider BMI at the age of 6 as a risk factor for later overweight. In accordance with our findings a recent observational study [50] demonstrated that overweight at the age of 5 years dwarfed all other risk factors for overweight at the age of 10 years.

### Potential Implication for Obesity Prevention

The most effective preventive measure would to reduce the proportion of children with a BMI >P75 at the age of 6 years [51]. For children with a BMI>P75 at the age of 6 years targeted preventions appear justified: already obese children have to be addressed with obesity treatment programs throughout youth because of a high persistence of obesity into adulthood in absence of interventions [44]. Although there are no evidence based guidelines yet as to the effectiveness of early interventions in overweight children at the age of 6 years these data point to the need for targeted preventive measures, since about half of them will not reach normal weight without an intervention. An appropriate strategy for children with high normal BMI values at age 6 years (P75<BMI≤P90) needs to be identified since about a third of them will become at least overweight. Targeting these children however, might be a challenge unless health care workers become aware of the considerable risk for overweight in children in the P75<BMI≤P90 category. Since age around school entry is shown as a critical period for staying or becoming overweight/obese school physicians and other health professions examining all children at school entry may play a key role to adopt preventive measures, which need to be targeted at those at risk: children with a BMI<P50 are not at high risk of overweight and might not need any intervention. This observation might question the need of universal prevention like school-based obesity prevention. There are only few studies showing small favourable effects on overweight/obesity (mainly in subgroups) [52], [53] but especially in overweight children [54]. Universal prevention addressing the entire pediatric population is useful to improve overall lifestyle behaviours but may not confer optimal resource allocation for obesity prevention [49], [53], [55], [56]. Our data point to the role of family physicians and pediatricians to advise parents about their children's risk for obesity and to initiate targeted prevention and provide guidance for counseling at school entry age.

### Strengths and Limitations

Based on the CART analysis the risk of overweight in adolescence in children in different BMI categories at the age of 6 can be predicted. Prediction is widely independent from the reference system: BMI cut-off values based on different populations (Kromeyer-Hauschild [30] and IOTF [40]). Therefore these findings appear to be transferable to different populations. The absolute risk within each BMI category, however, will be higher in populations with a higher overall risk for overweight/obesity as demonstrated.

Measurement of BMI was highly standardized in all cohorts and the cohorts are comparable with respect to the reported BMI values. While KOPS was representative for all children in Kiel attending the SEH [25], [26], DONALD was a convenience sample comprising families with an above average socioeconomic status. MAS is a population-based birth cohort in which children with an allergic family history were oversampled. Due to different study intentions for follow up the follow-up rates were high in DONALD and MAS but lower in KOPS. Losses to follow-up pertaining to lower educational status are likely and account for underestimation for the observed overall effects. [9] Unfortunately, we could not extend our study to early adulthood but it would be interesting to follow the cohorts until at least 21 years.

## Conclusions

Risk of overweight/obesity in adolescence can be predicted in children at the age of 6 years allowing for parent counselling or risk guided interventions. Targeted intervention programs (including prevention and therapy measures) during primary school age for high risk children need to be designed and their impact on overweight/obesity needs to be assessed.

## Supporting Information

Figure S1 **Classification tree for overweight/obese boys at the age of 14 years.** Classification tree for overweight/obese boys at the age of 14 years by different BMI categories based on percentiles created with the revised BMI LMS coefficients corresponding to the pooled international (IOTF) cut-offs at the age of 6, maternal obesity and education level. Prevalence of overweight/obesity in adolescence in each knot with 95% CI's.
(TIF)

Figure S2 **Classification tree for overweight/obese girls at the age of 14 years.** Classification tree for overweight/obese boys at the age of 14 years by different BMI categories based on percentiles created with the revised BMI LMS coefficients corresponding to the pooled international (IOTF) cut-offs at the age of 6, maternal obesity and education level. Prevalence of overweight/obesity in adolescence in each knot with 95% CI's.
(TIF)
