# Supplementary figures and images for: Overweight in Adolescence Can Be Predicted at Age 6 Years: A CART Analysis in German Cohorts
Source: PLoS One. 2014 Mar 27;9(3):e93581. doi: 10.1371/journal.pone.0093581 (PMC3968156; doi:10.1371/journal.pone.0093581)

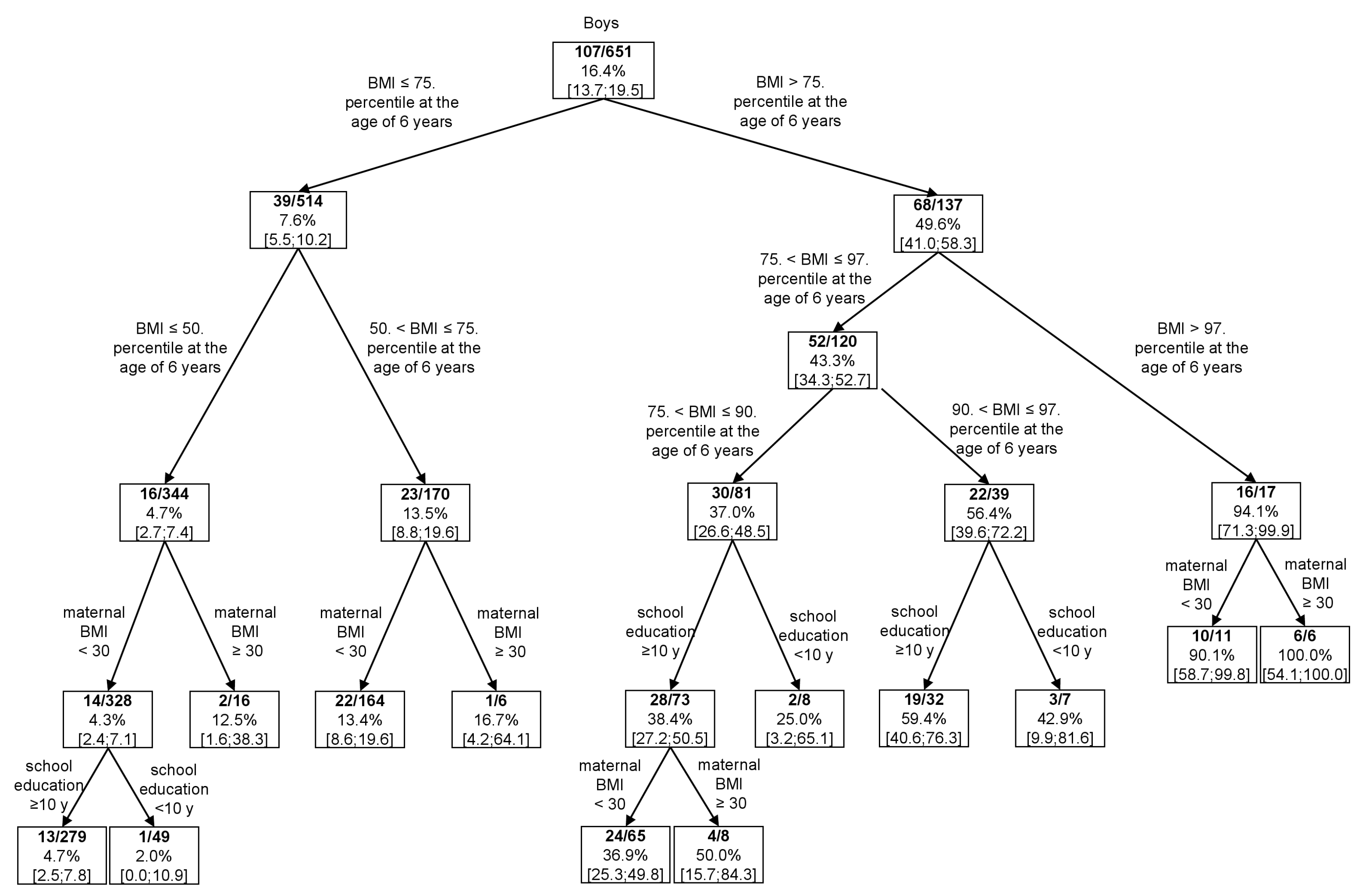

Supplement: Figure S1 — Classification tree for overweight/obese boys at the age of 14 years. Classification tree for overweight/obese boys at the age of 14 years by different BMI categories based on percentiles created with the revised BMI LMS coefficients corresponding to the pooled international (IOTF) cut-offs at the age of 6, maternal obesity and education level. Prevalence of overweight/obesity in adolescence in each knot with 95% CI's. (TIF) [file pone.0093581.s001.tif]

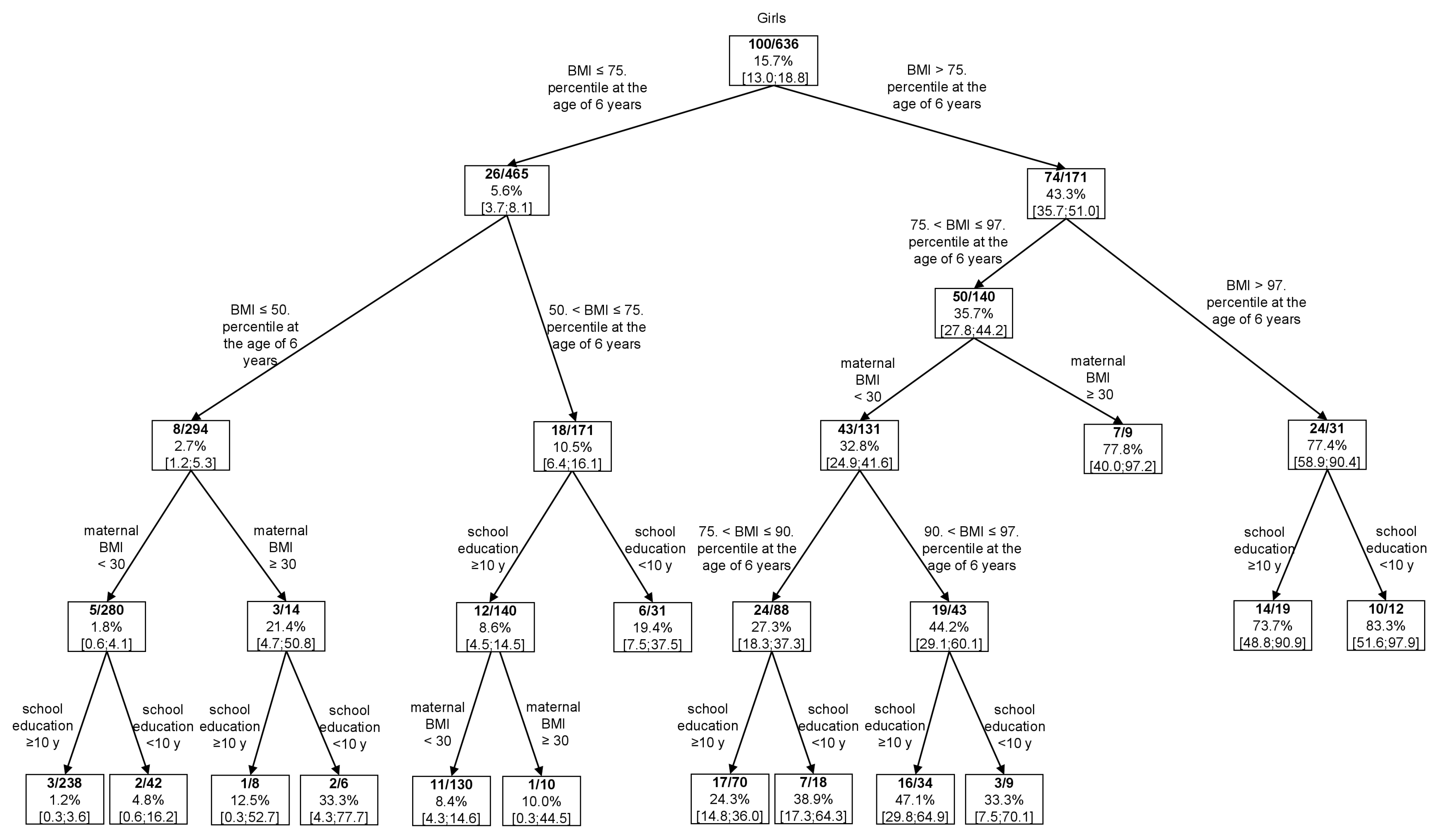

Supplement: Figure S2 — Classification tree for overweight/obese girls at the age of 14 years. Classification tree for overweight/obese boys at the age of 14 years by different BMI categories based on percentiles created with the revised BMI LMS coefficients corresponding to the pooled international (IOTF) cut-offs at the age of 6, maternal obesity and education level. Prevalence of overweight/obesity in adolescence in each knot with 95% CI's. (TIF) [file pone.0093581.s002.tif]
